# The Olympia anatomic polished cemented stem is associated with a high survivorship, excellent hip-specific functional outcome, and high satisfaction levels: follow-up of 239 consecutive patients beyond 15 years

**DOI:** 10.1007/s00402-021-03992-z

**Published:** 2021-07-25

**Authors:** Gareth S. Turnbull, Claire Marshall, Jamie A. Nicholson, Deborah J. MacDonald, Nicholas D. Clement, Steffen J. Breusch

**Affiliations:** 1 Department of Orthopaedics, The University of Edinburgh, 49 Little France Crescent, Old Dalkeith Road, Edinburgh, EH16 4SA UK; 2 Department of Orthopaedics, Royal Infirmary of Edinburgh, 51 Little France Crescent, Edinburgh, EH16 4SA UK; 3 University of Strathclyde, 16 Richmond St, Glasgow, G1 1XQ UK

**Keywords:** Anatomic, Stem, THA, Survivorship, PROMs, Polished, Cement

## Abstract

**Introduction:**

The Olympia femoral stem is a stainless steel, anatomically shaped, polished and three-dimensionally tapered implant designed for use in cemented total hip arthroplasty (THA). The primary aim of this study was to determine the long-term survivorship, radiographic outcome, and patient-reported outcome measures (PROMs) of the Olympia stem.

**Patients and methods:**

Between May 2003 and December 2005, 239 patients (264 THAs) underwent a THA with an Olympia stem in our institution. Patient-reported outcome measures were assessed using the Oxford Hip Score (OHS), EuroQol-5 dimensions (EQ-5D) score, and patient satisfaction at mean 10 years following THA. Patient records and radiographs were then reviewed at a mean of 16.5 years (SD 0.7, 15.3–17.8) following THA to identify occurrence of complications or revision surgery for any cause following surgery. Radiographs were assessed for lucent lines and lysis according to Gruen’s zones

**Results:**

Mean patient age at surgery was 68.0 years (SD 10.9, 31–93 years). There were 156 women (65%, 176 THAs). Osteoarthritis was the indication for THA in 204 patients (85%). All cause stem survivorship at 10 years was 99.2% (95% confidence interval [CI], 97.9%–100%) and at 15 years was 97.5% (94.6%–100%). The 15-year stem survival for aseptic loosening was 100%. Analysis of all-cause THA failure demonstrated a survivorship of 98.5% (96.3%–100%) at 10 years and 95.9% (92.4%–99.4%) at 15 years. There were 9 THAs with non-progressive lucent lines in a single Gruen zone and 3 had lines in two zones, and no patient demonstrated signs for lysis. At a mean of 10-year (SD 0.8, 8.7–11.3) follow-up, mean OHS was 39 (SD 10.3, range 7–48) and 94% of patients reported being very satisfied or satisfied with their THA.

**Conclusions:**

The Olympia stem demonstrated excellent 10-year PROMs and very high rates of stem survivorship at final follow-up beyond 15 years.

## Introduction

Excellent long-term outcomes have been demonstrated for cemented total hip arthroplasty (THA) performed with a variety of different femoral stem designs [1–3]. Whilst the majority of modern femoral stem designs are straight, normal femoral anatomy is curved when viewed in the coronal plane [4]. This mismatch predisposes to a recognized pattern of sagittal plane implant malalignment within the femoral canal, which results in a variation in the cement mantle thickness around the implant [5, 6].

**Fig. 1:**
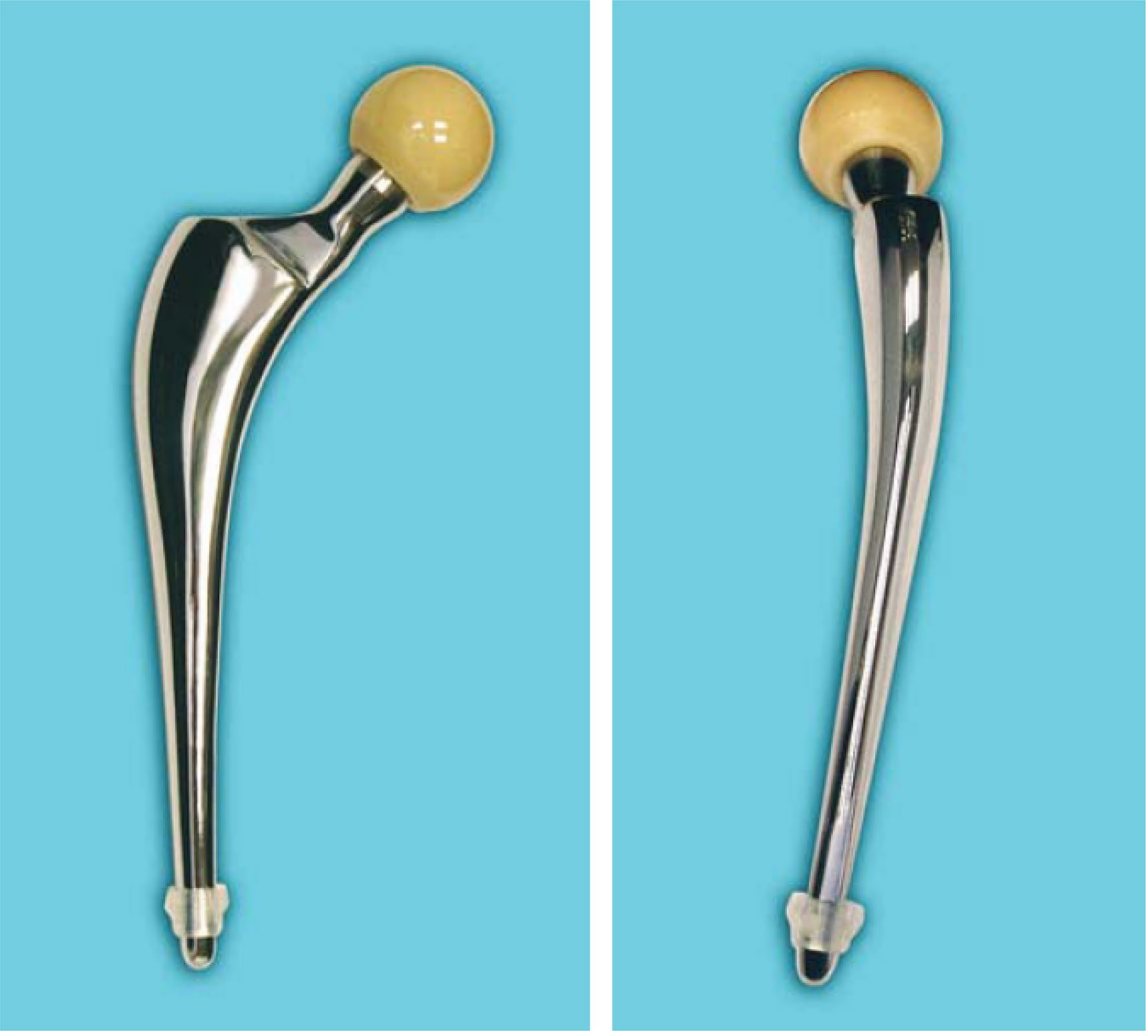
Orthogonal views of the Olympia stem, demonstrating anatomical shape in both planes [10]

To counter this, sided anatomic stems have been developed including the Olympia femoral stem (Biomet UK Ltd.). The Olympia is a stainless steel, highly polished, three-dimensionally (3D) tapered femoral stem with an anatomic shape in two plains (Fig. 1). Compared to straight stemmed designs such as the Exeter (Stryker Orthopaedics, Mahwah, NJ), anatomic stems are more reliable at producing a centralized and anatomical position within the femoral canal and maintaining a consistent cement mantle thickness around the implant [5, 7, 8]. Ultimately, this reduces the risk of cement mantle deficiency that can be associated with osteolysis and potential periprosthetic fracture [5, 7–9]. The Olympia has a number of other features aimed at maintaining cement mantle integrity. Tensile stresses and abrasion at the cement mantle are reduced by a highly polished stem surface (Ra 10 nm). A smooth cross-sectional design containing no edges further minimizes stress risers within the cement mantle. The 3D taper includes an oval metaphyseal section to improve rotational stability and implant centralization. A double reduced taper aims to limit subsidence, whilst a natural anteversion is built into the 3D design that mirrors the native femur [10].

There has been one mid-term study of the Olympia stem at 9 years with overall survival for aseptic loosening reported at 100%. However, there are limited published data on the survival and functional outcome of the stem after 10 years [10]. The aims of this study were to investigate the long-term survivorship (minimum of 15 years), radiographic outcome, and the associated patient-reported outcomes of the Olympia femoral stem in patients who underwent a primary total hip arthroplasty (THA).

### Patients and methods

Between May 2003 and December 2005, 264 consecutive primary THAs (239 patients) incorporating an Olympia stem were performed or supervised by the same surgeon within our institution. These patients were identified from a prospectively compiled arthroplasty patient database. In those who had received bilateral THAs, at least 6 months were required between consecutive operations for each THA to be analysed individually. If the time between THA operations in an individual patient was less than 6 months, PROMs were only sampled at follow-up from the last procedure performed to minimize the impact of surgical recovery from a contralateral THA on PROMs [11]. Two THAs were excluded as a result. No bilateral THAs performed simultaneously were included in this study.

Patient demographics and indication for THA were recorded and The Scottish Index of Multiple Deprivation used to assign social deprivation scores to patients based on postcode. The SIMD ranks geographic areas based on seven domains: income, employment, education, housing, health, crime, and geographical access. Data zones are defined by postcodes and once ranked nationally are divided into population-weighted quintiles with 1 representing the most deprived and 5 the least deprived [12].

Post-operative questionnaires including the following patient-reported outcome measures (PROMs) were sent to patients at a mean of 10.0 years (SD 0.8, 8.8–11.3) following THA: Oxford Hip Score (OHS), the EuroQol-5 dimensions (EQ-5D) score, and satisfaction scores. The OHS is a validated hip-specific outcome measure of 12 questions with five possible answers giving a score of between 0 and 48 [13]. Higher scores represent better function. The EQ-5D evaluates five domains of general health (mobility, self-care, usual activities, pain, and anxiety) and in addition a visual analogue scale (VAS) for general health [from 0 (worst) to 100 (best)] [14]. Pain was also assessed using a VAS [from 0 (worst) to 100 (best)]. Patient satisfaction was recorded by asking the patient “How satisfied are you with your hip?” The answer was documented using a 5-point Likert scale: very satisfied, satisfied, neither satisfied nor dissatisfied, dissatisfied, or very dissatisfied [15]. Patients were also asked in the questionnaire “Have you had any further surgery on or complications with the joint in question?” All patient notes and radiographs were reviewed again at a final mean of 16.5 years (SD 0.7, 15.3 to 17.8) following THA to examine for occurrence of any complications or surgery having occurred at any time following primary THA.

Radiographic analysis was performed on radiographs taken in the immediate post-operative period and during longer term follow-up as part of routine and unscheduled clinical follow-up. All radiographs were reviewed by a consultant radiologist and checked by a senior author or orthopaedic specialty trainee using PACS software and interpreted using the Gruen grading system [19]. Patients were searched on a national radiographic database at a mean of 16.5 years (SD 0.7, 15.3–17.8) following surgery to ensure that the results of the most recently available radiographs were included. Final radiographs included in the analysis were taken at a mean of 11.6 years following surgery (SD 3.3, 5 to 17.3).

### Surgical technique

All patients underwent surgery in a lateral decubitus position; an anterolateral or Modified Hardinge approach was used in all cases except one where a posterior approach was performed. After broaching and lavage of the femur, an Exeter cement restrictor was inserted. A third-generation cementing technique was performed involving pulsatile jet lavage, retrograde cement application, and 3-phase pressurization before Olympia stem insertion. A similar technique was used to implant a cemented acetabular component. Three cups were used, namely the Exeter Contemporary Flanged (CF) cup, the Hooded Exeter Low Profile (LP) cup, and the Charnley® Ogee Cup. All of the implants were fixed with Palacos R & G cement (Heraeus Kulzer GmbH; Heraeus Medical GmbH) with the exception of 3 patients who underwent THA with autogenous floor graft whose implants were fixed with Refobacin Palacos R (Heraeus Kulzer GmbH; Biomet Europe). THAs were routinely metal on polyethylene except for 2 that were ceramic on polyethylene. Each patient was administered 3 doses of antibiotics (1 dose preoperatively and 2 postoperatively) and DVT prophylaxis postoperatively.

### Statistical analysis

This was performed using Statistical Package for Social Sciences version 27.0 (SPSS Inc., Chicago, Illinois). Univariate analysis was performed using parametric (Student’s *t* test: paired and unpaired) and non-parametric (Mann–Whitney *U* test) tests, as appropriate, to assess continuous variables for significant differences between two groups. One-way analysis of variance (ANOVA) was used to compare continuous variables with multiple groups (OHS in Carstairs groups). Nominal categorical variables were assessed using a Chi-squared or Fisher’s exact test (if frequency less than 5 in one cell). Pearson’s correlation or Spearman’s rank correlation was used to assess the relationship between linear variables as appropriate. The Kaplan–Meier method was used to estimate the survival of the prosthesis. A *p* value of < 0.05 was considered significant in all analyses.

Ethical approval was obtained for this study. Collection of data was independent of the routine clinical care of the patient. Whilst the lead author was involved in developing the prospective database and original implant design, he remained independent from the process of data evaluation and manuscript preparation to exclude any potential source of bias.

## Results

### Patient demographics

Mean patient age at surgery was 68.0 years (SD 10.9, 31–93 years). There were 156 women (65%, 176 THAs). Osteoarthritis was the indication for THA in 204 patients (85%), rheumatoid arthritis in 14 patients (6%), and other diagnoses in 21 patients (9%). The mean body mass index (BMI) was 28.6 kg/m^2^ (range 17 to 45). Surgical implant variables are detailed in Table 1. At a mean of 10-year (range 6.4 -11.3) follow-up, there were 157 patients alive (66%, *n* = 157/239, 176 THAs) with 82 patients (34%, *n* = 82/239, 88 THAs) having died of unrelated causes. At a mean of 16.5-year (SD 0.7, 15.3 to 17.8) follow-up, there were 90 patients alive (38%, *n* = 90/239, 94 THAs) with 149 patients (62%, *n* = 149/239, 170 THAs) having died of unrelated causes.

**Table 1:** Breakdown of implant parameters used in patients

Olympia size	%	Cup size	%	Head size	%	Taper	%	Centraliser used	%
1	14.5	44/22	1.7	22 short metal	0.9	1214	96.6	Yes	51.3
2	21.4	43/28	5.1	22 medium metal	0.9	No data	3.4	No	48.7
3	29.1	44/28	0.9	28 short metal	17.1				
4	21.4	47/28	3.4	28 medium metal	54.7				
5	8.5	48/28	32.5	28 long metal	19.7				
6	1.7	50/28	26.5	28 short ceramic	1.7				
unknown	3.4	52/28	17.9	32 short metal	0.9				
		54/28	6.8	no data	4.3				
		56/28	0.9						
		56/32	0.9						
		no data	3.4						

### Implant survival and patient experience of complications

**Fig. 2:**
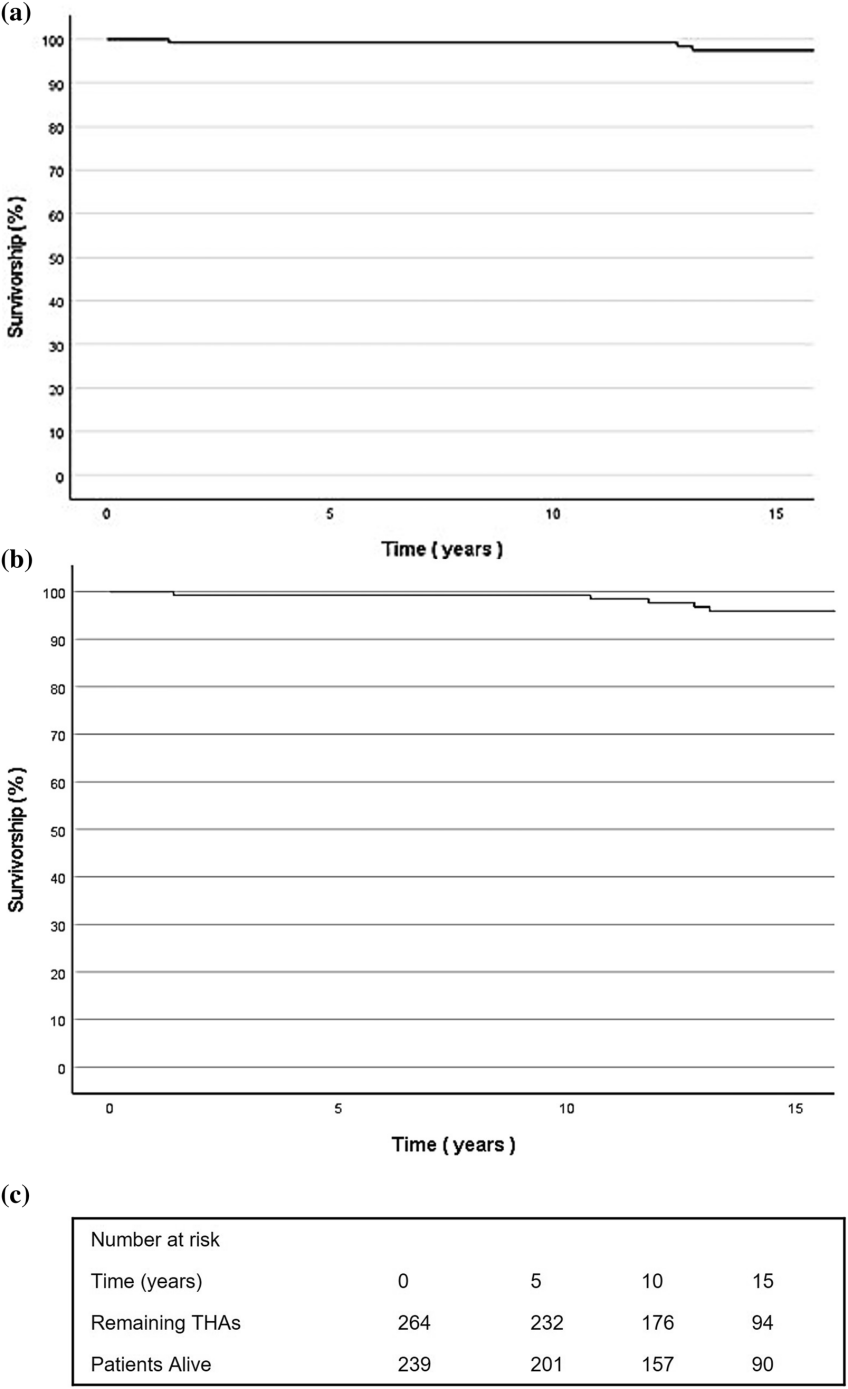
Kaplan–Meier analysis of the Olympia stem. **A** Survivorship of the Olympia stem over 15 years with end-point of stem revision for any reason. **B** THA survivorship over 15 years with end-point of THA failure for any reason. **C** Number of implants at risk at each time point also displayed

At a mean 16.5-year (SD 0.7, 15.3 to 17.8) follow-up, there were five revisions (5/264, 1.9%) in total. All-cause stem survivorship at 10 years was 99.2% (95% confidence interval [CI], 97.9%-100%) and at 15 years was 97.5% (95% CI 94.6%–100%) (Fig. 2A). The 15-year stem survival for aseptic loosening was 100%. Analysis of all-cause THA failure demonstrated a survivorship of 98.5% (95% confidence interval [CI], 96.3%–100%) at 10 years and 95.9% (95% CI 92.4%–99.4%) at 15 years (Fig. 2B). The 10-year survival for the end-point of re-operation for any reason was 98.1% (95% CI 97.3%–98.9%), and at 15 years, this was 91.7% (95% CI 91.2%–92.2%). Dislocation rate at 15 years was 0%, with no episodes identified on patient notes, radiographs, or on patient-reported feedback.

**Fig. 3:**
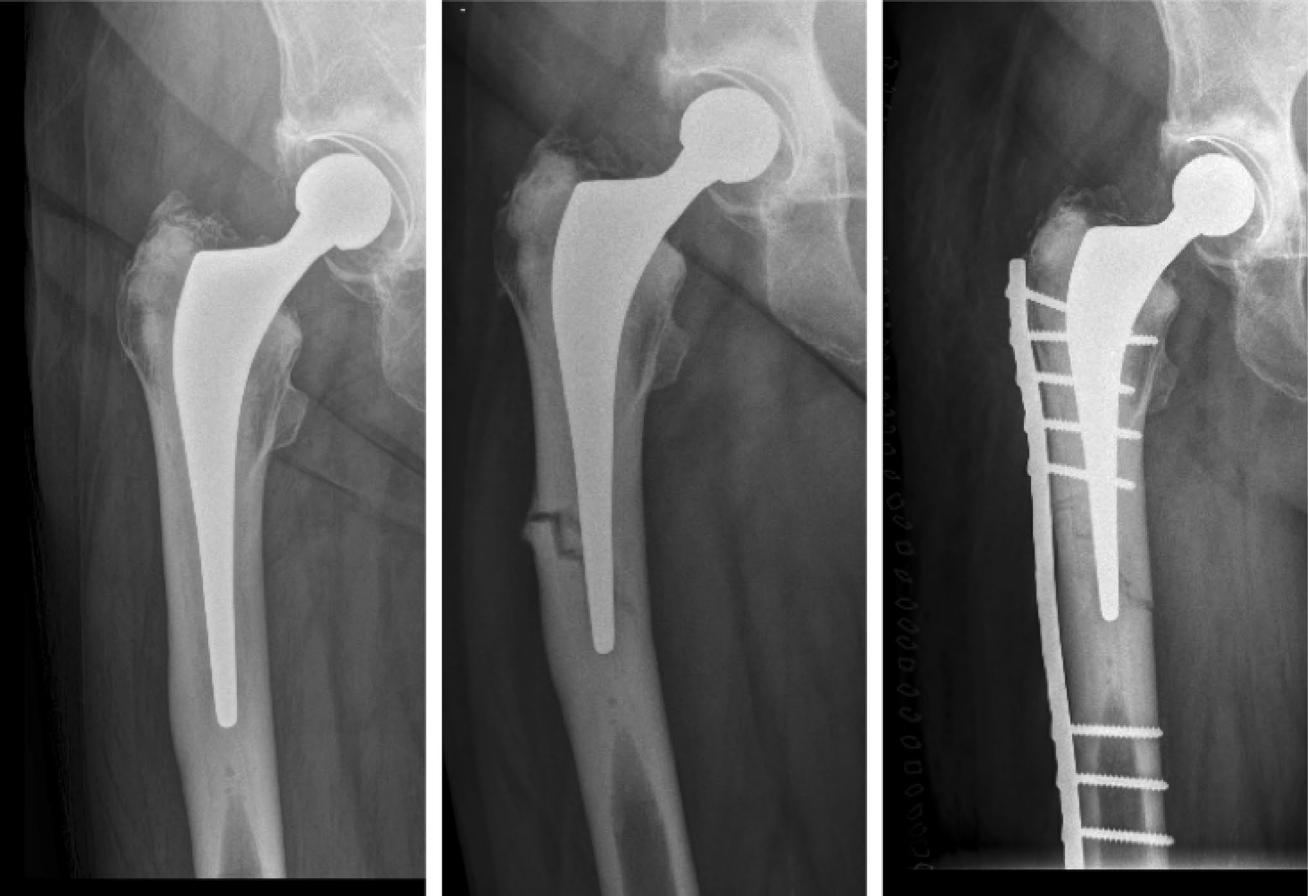
Development of an atypical periprosthetic fracture related to bisphosphonate usage in a patient with clear antecedent symptoms 13 years following index THA, with ORIF performed

Overall, two patients underwent revision for persistent deep infection; one patient underwent single-stage revision at 1 year following surgery, whilst the second patient underwent single-stage revision 13 years following THA. In both cases, revision was successful with no further surgery required and an infecting organism identified prior to revision. One patient sustained an atypical periprosthetic fracture requiring ORIF 13 years following index THA, related to bisphosphonate usage with clear antecedent symptoms and radiographic changes identified prior to development of periprosthetic fracture (Fig. 3). Two acetabular revisions were performed; one for aseptic loosening that developed following a heavy mechanical fall, with no stem revision required at 12 years following index THA; a further acetabular revision for aseptic loosening and implant migration was performed 11 years following index THA with cement-in-cement stem revision (original stem not found to have developed aseptic loosening) also performed.

Complications and reoperations were found to occur following 10 THAs with patient experience of complications detailed in Table 2. However, the majority of complications and reoperations (6/10, 60%) occurred more than 1 year following surgery.

**Table 2:** Complications identified following THA

Complication	Number	Comments
PE/DVT	1	Post-op PE and spontaneous DVT
Periprosthetic fracture	2	Vancouver A fracture treated conservatively in one patient Atypical fracture related to bisphosphonates revised in one patient
Infection	3	1 superficial infection debrided successfully 2 stems revised for deep infection
Aseptic acetabular loosening	2	Revision required in both cases, one developed following a fall
Persistent haematoma/soft-tissue dehiscence	2	One anticoagulated patient required haematoma washout. Attempted iliotibial band repair was performed in another patient 9 months post-op

### Radiological analysis

**Fig. 4:**
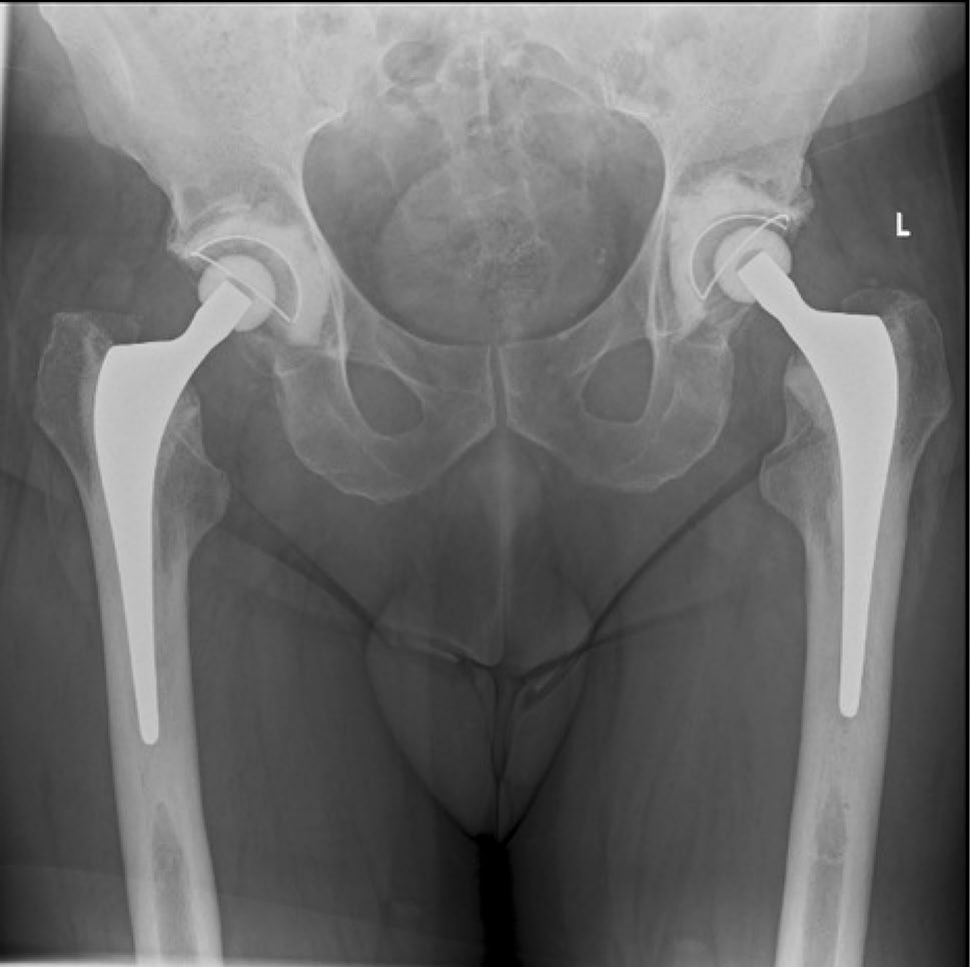
15-year follow-up radiographs for a patient that underwent staged bilateral THR for AVN at the age of 39, with primary autogenous acetabular impaction grafting performed due to marked cyst formation and sclerosis

There were radiographs available for 202 of the 239 patients (224 THAs) with final radiographs taken at a mean of 11.6 years following surgery (SD 3.3, 5–17.3) (Example in Fig. 4). Radiographs were not available for 37 patients (40 THAs) who died before routine follow-up or prior to the adoption of digitised radiographs in our institution. A total of 171 (77%) THA radiographs were reported as normal, whilst abnormalities were reported in the remaining 53 (23%) THAs as detailed in Table 3. Only 12 (5%) of the stems had lucent lines, all of which were non-progressive and noted to be clinically asymptomatic during clinical follow-up (Table 3). No stem demonstrated significant signs of lysis (lucent line > 2 mm). Analysis of immediate post-operative radiographs also found Barrack grade A cement mantles in all cases.

**Table 3:** Radiological analysis of implants

No. of THAs (%)	Radiograph comments	
171 (77)	Normal	
9 (4)	Heterotrophic ossification	
32 (14)	Features of acetabular demarcation	*DeLee and Charnley Zone Affected* Zone 1 – 22 (11) Zone 1 and 2 – 2 (1) Zone 1 and 3 – 2 (1) Zone 1, 2 and 3 – 3 (1.5) Zone 2 – 1 (0.5) Zone 3 – 2 (1)
12 (5)	Features of femoral stem demarcation	*Gruen Zone Affected* Zone 1 – 2 (1) Zone 1 and 7 – 2 (1) Zone 3 – 1 (0.5) Zone 4 – 2 (1) Zone 4 and 5 – 1 (0.5) Zone 7 – 4 (2)

### Patient satisfaction and functional outcome at 10 years

At a mean of 10-year (SD 0.8, range 8.7–11.3) follow-up, postal questionnaires were returned by 117 patients (76%) that underwent 134 THAs. 94% of patients reported being very satisfied or satisfied with their THA. Furthermore, 84% of patients were likely or extremely likely to be willing to undergo the same operation if needed at follow-up. Only two patients reported dissatisfaction, with the remaining 4% of patients neither satisfied nor dissatisfied with their THA at long-term follow-up. Patient age at surgery (*p* = 0.530), deprivation level (*p* = 0.355), and gender (*p* = 0.544) had no impact on satisfaction levels. However, patient experience of complications (*p* = 0.023, odds ratio [OR] 5.69, 95% CI 1.47–21.4) and a BMI ≥ 35 kg* m*^*−2*^ (*p* = 0.007, OR 1.55, 95% CI 1.06–2.66) were both associated with an increased risk of dissatisfaction.

The mean OHS reported was 39 (SD 10.3, 7–48). There were no correlations found between OHS and patient age (*p* = 0.275), gender (*p* = 0.889), BMI (*p* = 0.197), femoral stem size (*p* = 0 0.601), or head size (*p* = 0 0.724). However, a higher deprivation level (SIMD decile < 5) was associated with a significantly lower OHS, EQ-5D index, and inferior pain scores at follow-up (Table 4). Patients that experienced complications also reported a significantly lower OHS at follow-up (39.6 SD 10.6 v 30.5 SD 7.5, *p* = 0.018) and lower EQ-5D index score (0.84 SD 0.15 v 0.65 SD 0.09 *p* < 0.001). However, EQ-5D health (*p* = 0.73) and pain (*p* = 0.34) scores were not impacted by experience of complications or patient BMI (*p* > 0.05). Patients with a lower OHS were more likely to report lower satisfaction ratings at follow-up (*r* = 0.517, *p* < 0.001).

**Table 4:** Impact of deprivation on functional outcome at 10 years

	SIMD Decile < 5	SIMD Decile ≥ 5	*p* value
OHS	35.6 (10.4)	40.2 (11.0)	0.003
EQ-5D Index Score	0.79 (0.16)	0.86 (0.14)	0.016
VAS Pain Score	68.7 (32.8)	81.1 (25.3)	0.021

Results displayed as mean (standard deviation). *OHS* Oxford Hip Score, *EQ-5D* EuroQol-5 Dimension Questionnaire, *VAS* visual analogue scale, *SIMD* Scottish Index of Multiple Deprivation

## Discussion

The overall survival rate for the Olympia stem was excellent at a mean of 15-year follow-up, with high patient satisfaction levels and excellent OHS also demonstrated at a mean of 10 years. Survivorship, with revision of the stem for aseptic loosening as the end-point, was 100% at 15 years. Overall survival to revision for any reason was above 98% at 10 years and 96% at 15 years. There was a low rate of non-progressive radiolucent lines, with no cases of lysis, around the stem which supports continued longevity in the future. In addition, the stem when used as part of a THA was associated with excellent PROMs and high patient satisfaction, which was achieved irrespective of patient gender, age at operation, or BMI; however, social deprivation and complications were associated with lower PROMs.

A recent study reported the survival rate for all-cause revision of the Exeter™ stem (Stryker, Newbury UK) to be 91.2% at 13.5 years [16]. A further review reported Exeter stem 10-year survival rate at 97.8% for all-cause revision and 98.9% for aseptic loosening, with a mean OHS of 36 [17]. In a radiological analysis of the Olympia, we found that 6% of patients had signs of femoral demarcation at 10-year follow-up. In an analysis of the Exeter stem at a mean of 12.7 years, Hook et al*.* reported defects of the cement mantle (Barrack grade C and D) in 28% of stems and a revision rate for aseptic loosening and osteolysis of 1.1% [17]. The Olympia stem appears to compare favourably to the Exeter in terms of both revision rate and functional outcome measured by the OHS. When compared to another anatomic stem design, such as the *Lubinus SP II®* (Link, Hamburg, Germany), results were again favourable, with all-cause 10-year survival of the Lubinus SP II® reported as 98.3% [18].

The low incidence of periprosthetic fracture (PPF) demonstrated with the Olympia stem is particularly significant, with only one revision performed in this study, in a patient that developed the recognized phenomenon of an atypical PPF related to bisphosphonate usage [19]. Revision rates for PPF in straight stems such as the Exeter have been reported to be higher at 2.3% [20] and 1.5% for the updated Exeter V40 stem at 10 years [16]. The PPF rate of the Exeter was recently compared to another anatomic stem, the *Lubinus SP II®* (Link, Hamburg, Germany), and found to be ten times higher [21]. Minimizing the risk of PPF in patients has large clinical and socioeconomic implications. Patients revised for PPF display poor functional outcomes scores, often require resource intensive clinical management, and have a similar mortality rates when compared to neck of femur fracture patients up to 6 months post-injury and a reported 1-year mortality rate of 9.7% [22]. Future projections suggest that the number of periprosthetic fractures is expected to increase by 4.6% every decade over the next 30 years [23]. In this context, increased use of anatomic stems therefore seems a wise investment.

Socioeconomic status has previously been shown to negatively affect the functional outcome of primary and revision THA, at a national level and within our local population, with inferior joint-specific and health scores demonstrated [22, 24, 25]. In this respect, the Olympia stem appeared to have a similar outcome, with significantly lower OHS, EQ-5D index scores and increased pain scores reported in patients with increased social deprivation. However, deprivation level did not affect satisfaction at long-term follow-up. The Olympia stem appeared to perform equally well regardless of patient age or gender. Patient BMI also had limited impact on outcome within the confines of our study, with OHS, EQ-5D pain scores, and implant survivorship unaffected at follow-up. However, patients with a BMI ≥ 35 were on average “satisfied,” whereas patients with a BMI ≤ 35 were on average “very satisfied.” Conflicting reports exist regarding the effect of obesity on outcomes following primary THA, with some studies suggesting that high BMI limits post-operative function and satisfaction, and increases revision and complication rates [26].

The complication rate was low, with 1.7% of patients experiencing a complication or re-operation within 12 months of their THA. This compares favourably to the literature, with 3% of patients expected to experience a significant medical complication and 7.5% of patients reported to experience a significant surgical complication following THA [27]. Nevertheless, at 10-year follow-up patients that experienced a complication continued to report significantly lower OHS and EQ-5D index scores and were less likely to be satisfied with their operation.

No episodes of dislocation following THA were identified at 15-year follow-up, despite a detailed search of patient notes, radiographs, and a patient-reported questionnaire. This again compares favourably to the literature, with a meta-analysis of over 13 000 primary THAs and minimum 12-month follow-up reporting a dislocation rate of 3.23% for the posterior approach and 2.18% for the anterolateral approach [28].

There are some limitations to our findings. Radiographic analysis was incomplete as reported radiographs were not available for all patients. Patient-reported outcomes were performed at 10 years and not repeated at 15 years which may limit their findings. There are also inherent limitations to postal questionnaires. Preoperative PROMS were also not routinely collected or available from our institution prior to 2006 and so were not available for comparison. A number of patients were also lost to death. Surgical implantation was supervised or performed by a single experienced surgeon in a high-volume arthroplasty centre, potentially limiting external validity of results. Furthermore, survivorship analysis was performed on a relatively small number of implants when compared to that available for stems used in higher volume such as the Exeter stem [2–4].

The Olympia stem was demonstrated to be associated with an excellent 15-year survivorship. When revision of the stem for aseptic loosening is considered, 15-year survivorship is 100%. This survival rate is supported by the low rate of non-progressive radiolucent lines, with no cases of lysis, and indicates continued longevity of the stem. PROMs were excellent and compare favourably to other commonly used implants. Furthermore, it has a negligible rate of PPF compared to other polished taper slip stems, which should promote continued use of the implant in future as the clinical burden of PPFs continues to increase.
